# An Improved Method for Stable Feature Points Selection in Structure-from-Motion Considering Image Semantic and Structural Characteristics

**DOI:** 10.3390/s21072416

**Published:** 2021-04-01

**Authors:** Fei Wang, Zhendong Liu, Hongchun Zhu, Pengda Wu, Chengming Li

**Affiliations:** 1 College of Geodesy and Geomatics, Shandong University of Science and Technology, Qingdao 266590, China; wfxs946773243@163.com (F.W.); sdny_xaa@163.com (H.Z.); 2 National Engineering Laboratory for Integrated Aero-Space-Ground-Ocean Big Data Application Technology, Xi’an 710072, China; lzdgis08@126.com (Z.L.); wupd@casm.ac.cn (P.W.); 3 Institute of Cartography and Geographic Information System, Chinese Academy of Surveying and Mapping, Beijing 100830, China

**Keywords:** 3D reconstruction, oblique images, feature point selection, image semantic and structural characteristics, two-tuple classification model

## Abstract

Feature matching plays a crucial role in the process of 3D reconstruction based on the structure from motion (SfM) technique. For a large collection of oblique images, feature matching is one of the most time-consuming steps, and the matching result directly affects the accuracy of subsequent tasks. Therefore, how to extract the reasonable feature points robustly and efficiently to improve the matching speed and quality has received extensive attention from scholars worldwide. Most studies perform quantitative feature point selection based on image Difference-of-Gaussian (DoG) pyramids in practice. However, the stability and spatial distribution of feature points are not considered enough, resulting in selected feature points that may not adequately reflect the scene structures and cannot guarantee the matching rate and the aerial triangulation accuracy. To address these issues, an improved method for stable feature point selection in SfM considering image semantic and structural characteristics is proposed. First, the visible-band difference vegetation index is used to identify the vegetation areas from oblique images, and the line feature in the image is extracted by the optimized line segment detector algorithm. Second, the feature point two-tuple classification model is established, in which the vegetation area recognition result is used as the semantic constraint, the line feature extraction result is used as the structural constraint, and the feature points are divided into three types. Finally, a progressive selection algorithm for feature points is proposed, in which feature points in the DoG pyramid are selected by classes and levels until the number of feature points is satisfied. Oblique images of a 40-km^2^ area in Dongying city, China, were used for validation. The experimental results show that compared to the state-of-the-art method, the method proposed in this paper not only effectively reduces the number of feature points but also better reflects the scene structure. At the same time, the average reprojection error of the aerial triangulation decrease by 20%, the feature point matching rate increase by 3%, the selected feature points are more stable and reasonable.

## 1. Introduction

The structure from motion (SfM) technique has been successfully used for the large-scale 3D scene reconstruction of oblique aerial images [1,2,3,4]. This process generally consists of three main steps: feature matching, aerial triangulation and dense multi-view stereo reconstruction [5,6]. Among them, feature matching is the first and foundation step, which directly affects the accuracy of subsequent tasks [7,8]. Classical algorithms such as the scale-invariant feature transform (SIFT) and speeded up robust features (SURF) algorithms are usually used to extract and describe feature points [9,10]. However, there exist two weaknesses in the process of large-scale oblique photography 3D reconstruction: (1) the number of feature points extracted by the above algorithm is very large, and a single image may include hundreds of thousands of feature points, which greatly reduces the efficiency of image matching and easily leads to the failure of aerial triangulation; (2) the quality of the feature points is uneven, and the feature points with poor stability drag down the matching rate and aerial triangulation accuracy. Therefore, selecting feature points with strong robustness and high stability in the feature matching stage to accelerate the matching speed and improve the matching quality is essential, and it has always been a difficult problem in this field [7,11]. Generally, feature point selection methods can be classified into two categories: single-scale selection methods and multiscale selection methods. The former takes feature points on a single scale as the research object and uses machine learning to classify and select feature points, such as bag-of-words models, vocabulary trees, and neural networks [12,13,14,15,16]. However, the feature point selection effect of this type of method depends on the quality of the sample set, and the sample set needs to be established separately for different scenarios, limiting their applicability in time-constrained real conditions. Therefore, in practical applications, more scholars choose the latter methods to automatically select the feature points [17,18,19], that is, selecting feature points based on a Difference-of-Gaussian (DoG) pyramid. This article also belongs to an improvement of this type of method.

In 2013, Wu [20] performed a systematic analysis of the feature points in the DoG pyramid and pointed out that those located at the higher level of the pyramid represent the image features well and can cover a relatively wide range. Therefore, Wu proposed the “preemptive feature matching” algorithm, which uses a small number of high-scale features for feature matching. This method greatly improves the image matching speed and reduces the time complexity from *O(n^4^)* to *O(n)*, and it is widely used because of its usability and effectiveness. Shah et al. [19] constructed a parallelized incremental SfM algorithm in which the preemptive feature matching approach was used for feature matching. Braun et al. [21] used this feature point selection algorithm to improve the building reconstruction rate and image matching quality when using oblique photography data for building construction process monitoring. Nex et al. [22] also used this algorithm to ensure the computation efficiency of large-scale image matching in the study of real-time mapping of building damage based on oblique photography. However, due to the significant difference in the viewing angle of the oblique images, there is a large perspective geometric distortion between the images. An existing feature point selection algorithm cannot obtain enough stable and reliable corresponding features, resulting in the lower matching rate and the aerial triangulation accuracy. To address this problem, an improved method for stable feature point selection in SfM considering image semantic and structural characteristics is proposed. The purpose and motivation of the proposed method is to improve the feature matching quality while ensuring the feature matching efficiency by mining higher-level stable features of the image.

The innovation and contributions of this work are summarized as follows:

- Aiming at the insufficient consideration of the stability and spatial distribution of feature points in existing methods, a new feature points selection method considering the semantic and structural characteristics of the image simultaneously is proposed to improve the stability and reliability of the selected feature points.
- A two-tuple classification model is constructed and a progressive multi-scale selection algorithm is developed, the new model and algorithm not only ensures the feature matching efficiency but also extract more reasonable feature points, which can better reflect the scene structure, and reduce the average reprojection error and improve the feature point matching rate.

The remainder of this paper consists of the following four sections: Section 2 presents the common and advanced feature point selection algorithm, and the limitations are discussed; Section 3 introduces the improved feature point selection method; Section 4 describes a series of experiments conducted to validate the reliability and superiority of the proposed method. The conclusions and future work are given in Section 5.

## 2. Related Work

### 2.1. Existing Feature Point Selection Method for Feature Point Selection

Feature point selection plays a crucial role in the process of 3D reconstruction based on the SfM technique, because the selected points are not only used to recover the imaging geometry orientation for image matching, but are also used to perform feature matching for the subsequent aerial triangulation [23]. Although computation efficiency of imaging matching can be greatly improved by introducing the sequence of the images or Global Navigation Satellite System (GNSS) data, feature matching is still a bottleneck because typical oblique images contain several thousands of feature points [24,25,26], which is the aspect we try to handle.

**Figure 1:**
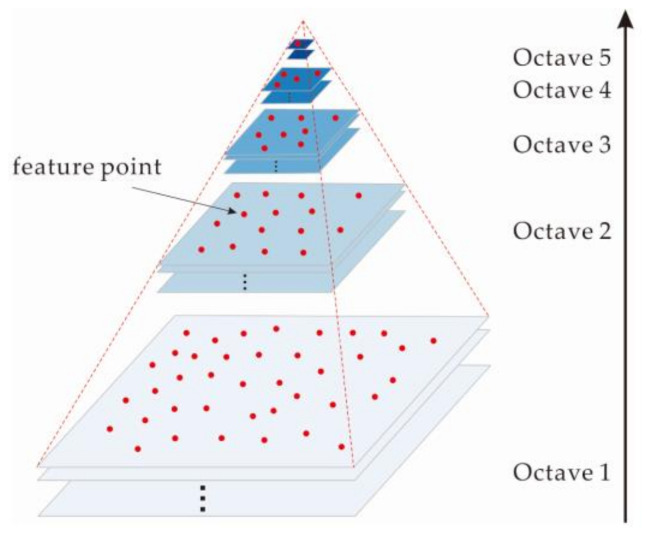
DoG pyramid.

A classic solution is to select a certain number of robust and stable feature points from the original feature points for feature matching. In current studies, Wu [20] proposed a common and practical method called the preemptive feature matching algorithm. In this method, only the higher-scale features of the DoG pyramid are selected for feature matching, which greatly increases the speed of feature matching by avoiding full pairwise matching. The detailed procedures of this method are as follows:

(1) A convolution with a Gaussian filter is performed on each image to construct a DoG pyramid. The features corresponding to each octave are then extracted as local extreme values in the image and across scales, as shown in Figure 1.
(2) The DoG pyramid is traversed level by level (from top to bottom) to select a small number of feature points from each image for image-pair selection. For example, selecting the first 100 feature points for each image, if there are 4 or more feature point matches among the 100 feature points of two images, these two images are deemed to form an image pair.
(3) A number threshold (*T_n_*) is further determined for the feature point matching of an image pair.
(4) Suppose the level of the pyramid is *l* = {*l*_1_, *l*_2_, …, *l_n_*}, where level ln is the top level and number (*l_i_*) refers to the number of feature points in level *l_i_*. For each pair of images, their feature points are collected from top to bottom until $\sum \mathrm{number}\left(l_{i}\right)\;\geq {\;T}_{n}.$ Based on a large number of experiments, Wu [20] suggested that a threshold of 8192 is sufficient for most scenarios.
(5) The selected feature points are used for feature matching and are used as the input data for the subsequent aerial triangulation process.

### 2.2. Limitations of the Existing Method

During the reconstruction of large 3D scenes from oblique aerial images, the selected feature points play a crucial role in the following three aspects: (1) The number of selected feature points determines the computational efficiency of the feature matching process. (2) The quality of the selected feature points affects the feature matching rate and the aerial triangulation accuracy. (3) The distribution of the selected features determines the spatial structure of the sparse point cloud. For the current feature selection method, the features are constrained by their total number and Gaussian scale, which considerably improves the computational efficiency (aspect (1)). However, when the number of feature points used for matching is determined, points with strong stability and obvious structural features should be selected as often as possible. Some feature points which are not stable and have weak structural features are still selected by the existing method, resulting in the selected feature points not reflecting well in the scene structure and the feature matching rate and aerial triangulation accuracy cannot be guaranteed (aspect (2) and (3)).

**Figure 2:**
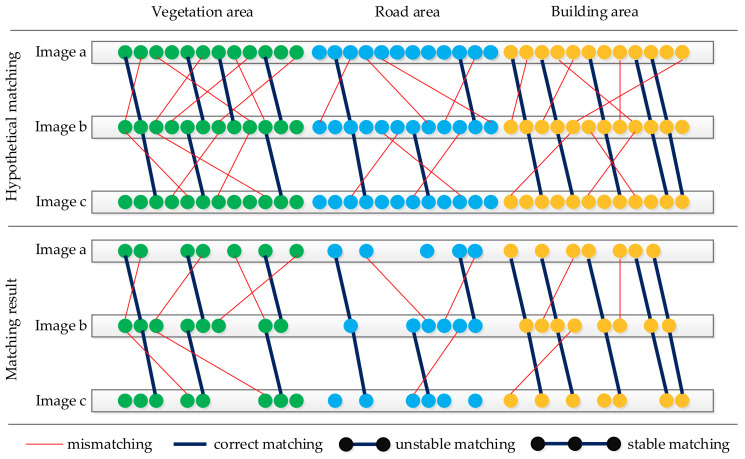
Limitations of the existing feature selection method.

Previous studies [13,23,27] have shown that certain feature points on vegetation, or on the road surface are not easily matched successfully, and points like those on the build structure are more favorable to match, because they are stable when seen from different viewpoints. This conclusion is also proven by numerous experiments with our actual oblique image data, and Figure 2 is a schematic diagram to describe the general experimental result of the existing feature point selection method. Although the number of feature points is successfully reduced by the existing method, there are still a certain number of mismatches and unstable feature points with fewer than two repeated matches in the selected feature points, especially in the vegetation area. The classic RANSAC algorithm [28] can be used to eliminate the mismatches between an image pair, but it is not very helpful to extract the stable feature points among many related image pairs. In addition, the spatial distribution of the feature points is random, which makes it difficult to accurately describe the scene structure.

## 3. Methodology

The key to solving the limitation of existing methods is to extract stable feature points in the image as much as possible. Traditional feature points acquired based on multiple invariant feature descriptors is not enough to guarantee effective matching and higher-level stable features of the image need to be mined. Hence, based on the quantitative and hierarchical parameters in the existing method [20], the vegetation and line features are selected as two new parameters for the feature point filtering in this paper. The reasons for the selection of the new parameters is that (1) these two parameters can accurately and conveniently depict the deep semantic and structural characteristics of the image [4,13]; (2) the feature points that can be described by these two parameters have been proved to be more suitable for the feature matching [4,5,11,27].

An improved method for stable feature point selection in SfM considering image semantic and structural characteristics is proposed, which mainly consists of three steps. (1) Recognition of the vegetation areas and the extraction of line features: the visible-band difference vegetation index (VDVI) is used to identify vegetation areas from an oblique image, while an optimized line segment detector (LSD) is used to extract line features. (2) Construction of the two-tuple classification model: the vegetation area recognition result is used as the semantic constraint, and the line feature extraction result is used as the structural constraint, establishing a feature point classification model and dividing feature points into three types. (3) Feature point progressive selection: the number threshold for feature point selection is determined, and feature points in the DoG pyramid are selected by classes and levels until the number of feature points is satisfied.

### 3.1. Recognition of Vegetation Areas and Extraction of Line Features

(1) Recognition of vegetation areas

A vegetation area is a typical weak texture area; that is, there are no significant texture features, such as corners and borders, in the vegetation area, the values of adjacent pixels are very close, and the differences among texture features are not obvious. The weak textures of the vegetation area leads to the poor stability of the feature points in it. Therefore, our method first identifies the vegetation area in the image.

The reflection spectrum of green vegetation in the visible light band has the characteristics of strong blue and red absorption and a strong green reflection. Researchers in vegetation remote sensing have proposed a large number of mature indexes [29,30,31] for calculating vegetation areas based on the above characteristics, such as the red-green ratio index (RGRI), excess green (EXG), and VDVI. The extraction accuracy of the VDVI is higher than those of the RGRI and EXG, reaching more than 90% [32]. Therefore, this paper uses the VDVI to extract vegetation information, and the mathematical formula is shown in Equation (1).

(1) $$\mathrm{VDVI}\;=\;\frac{2G\;-\;R\;-\;B}{2G\;+\;R\;+\;B}$$

**Figure 3:**
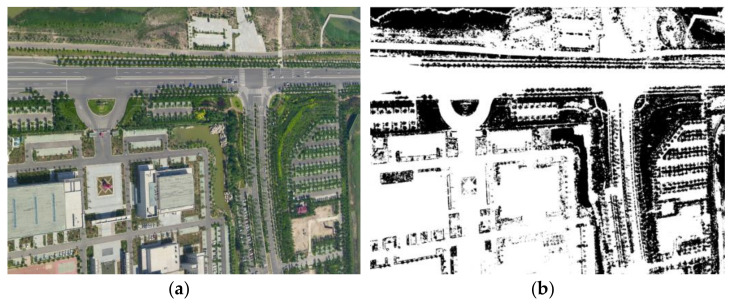
Recognition result of vegetation area. (**a**) Original oblique image and (**b**) recognition result of the vegetation areas (black parts, the gray value in the binary image is 0).

As depicted in Figure 3, the vegetation areas are successfully recognized from the original oblique image.

(2) Extraction of line features

**Figure 4:**
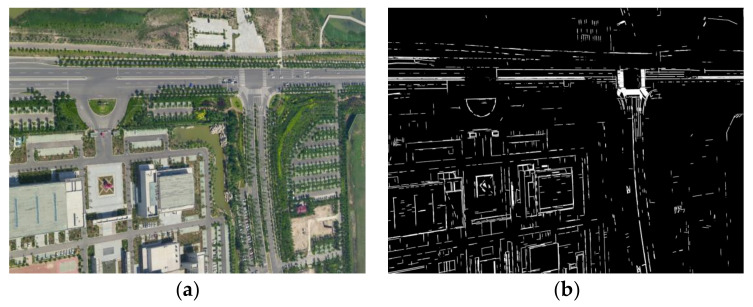
Extraction result of line feature. (**a**) Original oblique image and (**b**) extraction result of the line features (white parts, the gray value in the binary image is 255).

During 3D reconstruction, feature points that are located in areas that have distinct structures or textural information (such as building boundaries) are more stable than feature points located in other areas. Therefore, the LSD [33] was used to extract line features from the oblique images. LSD is a line segment detection and segmentation algorithm that has a fast detection speed, a high detection accuracy (subpixel level), a low false detection rate, and strong adaptability to different images. It includes six key steps: image scaling, gradient calculation, gradient pseudo-ordering, gradient threshold, region growth and rectangular approximation. The specific steps of this algorithm are detailed in the paper by [33] and will not be repeated in this article. An example is shown in Figure 4.

**Figure 5:**
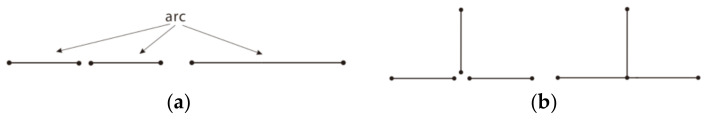
Connecting line segments. (**a**) Two separated nodes and their connection result and (**b**) three separated nodes and their connection result.

Since the line segments obtained by the LSD algorithm are scattered and discontinuous, this article optimized this method by converting these lines into vector data and constructing a node-arc topology [34] to connect the line segments, in which an arc refers to a line segment, forming by a start-node and an end-node. As shown in Figure 5, arcs are separated from each other. For two or more adjacent nodes, if the distance between them is less than the threshold value, the separated arcs will be aggregated. Specifically, if two arcs are parallel and collinear, their endpoints are connected first; if two arcs are parallel but not collinear, then they extend in a parallel direction. The spatial relationship between the arcs is calculated using the vector cross product [35].

**Figure 6:**
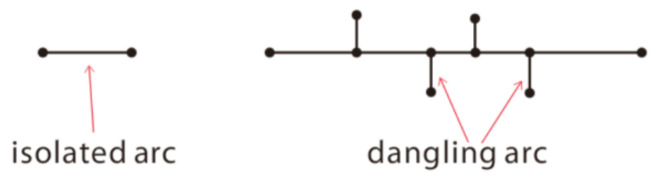
Isolated arcs and dangling arcs.

The topology is then refreshed, and the isolated and dangling arcs [36,37] that are separated by distances smaller than length threshold value are eliminated, as shown in Figure 6. In an actual computation, the threshold is automatically determined based on statistical principles, that is, the difference between the average value of the length of all arcs and the standard deviation of the length of all arcs.

### 3.2. Construction of the Two-Tuple Classification Model

The oblique image is first divided into two classes, namely, vegetation and non-vegetation areas, based on the VDVI. The classification threshold (CT) is automatically calculated according to the distribution of the vegetation index using Otsu’s method [38]. For any pixel, if VDVI > CT, then the pixel belongs to a vegetation area; if VDVI ≤ CT, then the pixel belongs a non-vegetation area.

The actual value range of VDVI is [–1, 1]. For the convenience of explaining and describing the image classification principle through VDVI, we suppose that the VDVI values of an image are in the range of [1, 2, …, L], and the number of pixels at level *i* is denoted by *n_i_*. The total number of pixels in an image is given by *N* = *n*_1_ + *n*_2_ + … + *n_L_*. Then, the probability distribution of each VDVI value is *p_i_* = n*_i_*/*N*. Now, suppose the oblique image is divided into two classes, *C*_0_ and *C*_1_, by a threshold at level k. *C*_0_ denotes the depths of levels [1, …, *k*], and C_1_ denotes the depths of levels [*k* + 1, …, *L*]. Then, the between-class variance is given by:

(2) $$\sigma_{B}^{2}{\;=\;\omega }_{0}{{(u}_{0}-u_{T})}^{2}{+\omega }_{1}{{(u}_{1}-u_{T})}^{2}{\;=\;\omega }_{0}\omega_{1}{{(u}_{1}{\;-\;u}_{0})}^{2}=\frac{{{[u}_{T}\omega \left(k\right)\;-\;\mu \left(k\right)]}^{2}}{\omega \left(k\right)(1\;-\;\omega \left(k\right))}$$

where *ω*_0_ and *ω*_1_ are the proportions of the image pixels accounted for by *C_0_* and *C_1_*. *μ*_0_ and *μ*_1_ are the mean values of *C*_0_ and *C*_1_. $u_{T}=\overset{L}{\underset{i=1}{\sum }}ip_{i}$, $\mu \left(k\right)=\overset{k}{\underset{i=1}{\sum }}ip_{i}$ and $\omega \left(k\right)=\omega_{0}$.

The optimal threshold, k*, is calculated by Equation (3).

(3) $$\sigma_{B}^{2}\left(k^{*}\right){\;=\;\max}_{1\;\leq \;k\;<\;L}\sigma_{B}^{2}\left(k\right)$$

Similarly, the image can be divided into line-feature and non-line-feature areas, depending on whether the pixels are located within the buffer zones of the linear features. Specifically, the buffer zone of the linear feature refers to a polygonal area obtained by expanding the line with a certain width on both sides.

Based on the semantic (vegetation or non-vegetation) and structural (line-feature or non-line-feature) information in the image, a two-tuple classification model for feature points is constructed and expressed as:

(4) $$f\left(i\right){\;=\;\alpha x}_{1i}{\;+\;\beta x}_{2i}\;i\;=\;1,2,\ldots ,N\;$$

where *f*(*i*) is the classification value of feature point *i*, *x*_1*i*_ is the semantic variable (Equation (5)), *x*_2*i*_ is the structural variable (Equation (6)), and *α* and *β* are the index weights, whose values range within [0, 2], and *α* + *β* = 2.

(5) $$x_{1i}\;=\;\left\{\begin{array}{c} {0,\;\mathrm{if}\;\mathrm{VDVI}}_{i\;}>\;\mathrm{CT}\;\\ {1,\;\mathrm{if}\;\mathrm{VDVI}}_{i}\;\leq \;\mathrm{CT} \end{array}\right.$$

(6) $$x_{2i}=\left\{\begin{array}{c} 1,\;\mathrm{if}\;\mathrm{BZFL}\;\mathrm{contains}\;i\;\\ 0,\;\mathrm{if}\;\mathrm{BZFL}\;\mathrm{does}\;\mathrm{not}\;\mathrm{contain}\;i \end{array}\right.$$

where BZFL is the buffer zone of a feature line.

The feature points in each level of DoG pyramid are thus classified into three types, as depicted in Table 1.

### 3.3. Feature Point Progressive Selection Algorithm

A progressive selection algorithm is proposed to ensure that high-quality feature points can be selected. In this algorithm, the DoG pyramid is constructed first, and the threshold for the number of feature points is then determined. Feature points are then selected level by level from the DoG pyramid until the number of feature points is satisfied. The procedures of this process are as follows.

Step 1: Construction of the DoG pyramid and label each level as *l* = {*l*_1_, *l*_2_, …, *l_n_*}, where level *l_n_* is the top level.

Step 2: Determine the threshold for the number of selected features. The position of the camera and the scene structure corresponding to the adjacent oblique images change very little. Therefore, referring to the existing literature, a general threshold for the number of features can be used, such as 8192 feature points.

Step 3: Construction of the two-tuple classification model. Recognize vegetation areas, extract line features of each oblique image, and construct the two-tuple classification model to calculate the classification value of each feature point in the DoG pyramid.

Step 4: Feature point classification. Classify the feature points into Types I, II and III based on the classification threshold in Table 1.

Step 5: Progressive selection: (1) On the premise that all the feature points of each level are retained, the number of levels required to meet the number threshold is calculated and recorded as *l_t_*; (2) in the range of the *l_t_*–*l_n_* level, the impact of hierarchical features on the quality of the feature points is considered to have a higher priority, Type I and II feature points are retained, and Type III feature points are eliminated; (3) from the *l_t−_*_1_ level, the impact of land-cover semantic and structural information is considered to have a higher priority, and only Type I feature points are retained; (4) after the selection in *l_t−_*_1_, calculate whether the total number of selected feature points satisfies the threshold. If it is satisfied, the traversal ends; if not, continue to look for the Type I feature points at the next level until the number threshold is satisfied or the *l*_1_ level is reached.

## 4. Results

### 4.1. Experimental Data and Computational Environment

**Figure 7:**
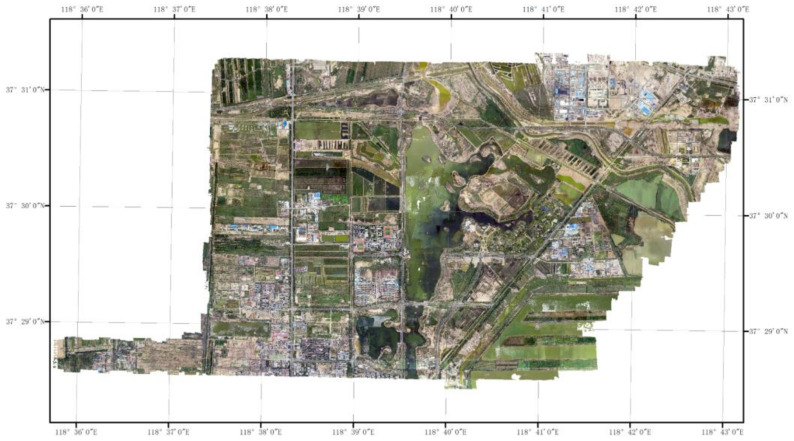
Experimental area.

The method proposed in this paper was embedded into the NewMap-IMS software, which is an independently developed reality modeling software by the authors in Chinese Academy of Surveying and Mapping. The experimental area is a 5.2 km × 7.8 km urban built-up area in Shandong Province, China. A 5-lens (1 vertical-view lens + 4 side-view lenses) UltraCam Osprey Prima (UCOp) camera was used in 29 flights and collected 11,795 images, which amounted to 2.08 TB of data. The details of the oblique image data are shown in Table 2, and the layout of the study area is illustrated in Figure 7. The operating environment was a Windows 10 64-bit operating system with an Intel Xeon Gold 6132 CPU at a main frequency of 2.6 GHz and with 200 GB of memory.

### 4.2. Qualitative Evaluation and Analysis

**Figure 8:**
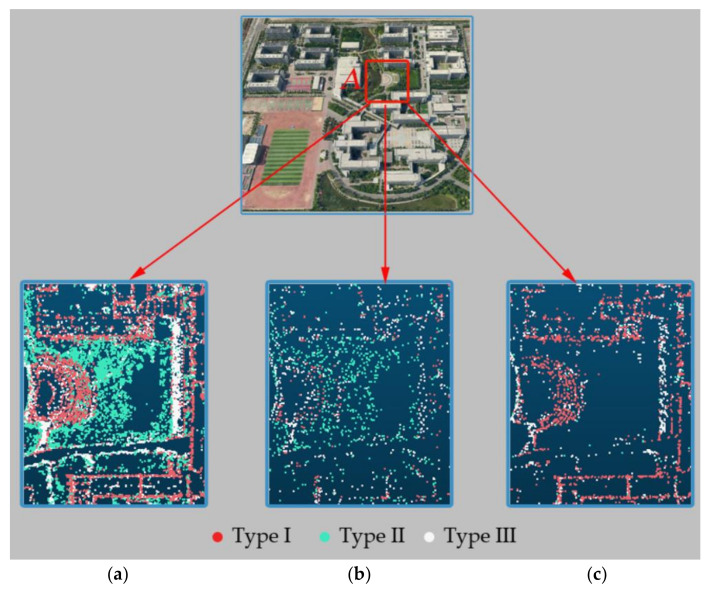
Sparse point cloud of a vegetation area (rectangle A) in an oblique image. (**a**) The result of the original SIFT algorithm, (**b**) the result of Wu method and (**c**) the result of the proposed method.

**Figure 9:**
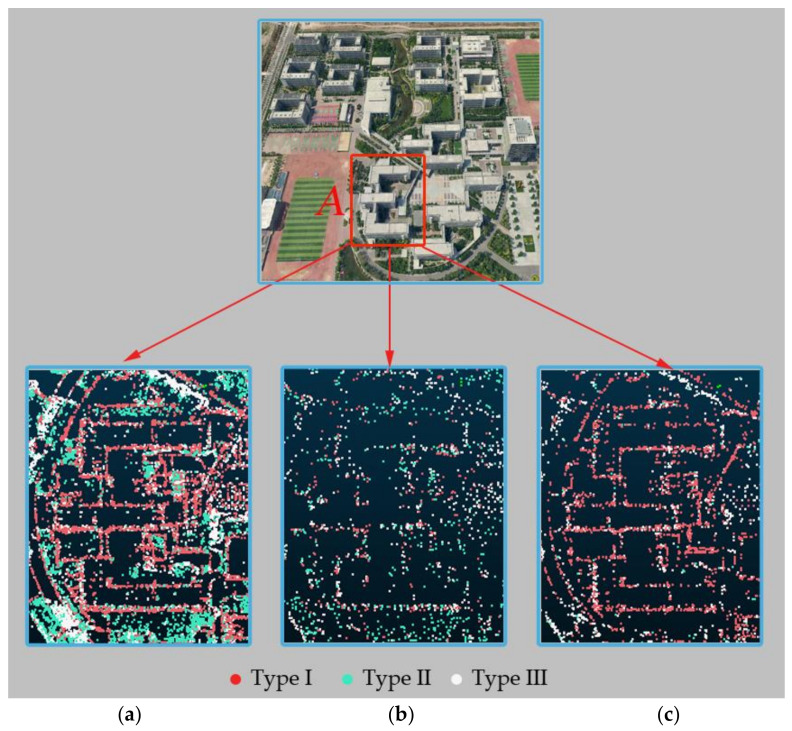
Sparse point cloud of the building area (rectangle A) in an oblique image. (**a**) The result of the original SIFT algorithm, (**b**) the result of Wu method and (**c**) the result of the proposed method.

The feature points extracted by the original SIFT algorithm [9], those selected by the method proposed by Wu (hereafter called the Wu method) [20], and those selected by the proposed method in this paper were respectively used input data for aerial triangulation to generate the sparse point cloud of the scene. Figure 8 and Figure 9 shows the sparse point cloud construction results of the three methods for a certain image in the experimental area.

Figure 8a–c demonstrate the results of the three methods in vegetation areas. The number of tie points generated by the original SIFT algorithm is the largest, and some of them are located in the vegetation area (Figure 8a). After feature point selection by the Wu method, the number of tie points was significantly reduced, and most of the tie points located in the vegetation area were removed. However, the distribution of tie points in the sparse point cloud is relatively irregular, which makes it unable to accurately describe the scene structure, and the structural characteristics of the ground object became blurred (Figure 8b). For the sparse point cloud construction results by the method proposed in this paper, tie points in the vegetation area were further reduced, and the tie clouds were concentrated in areas with obvious structural characteristics, such as boundary objects and building structure lines, showing that the method proposed in this paper can better characterize the scene structure with a small number of feature points (Figure 8c).

Figure 9a–c illustrate the results of the three methods in the building area. Similar to the sparse point cloud of the vegetation area, the number of tie points generated by the original SIFT algorithm is the largest, the number of points generated by the Wu method is the smallest, and the number of points generated by the method proposed in this paper is between them. A building is a ground object with obvious structural features. If the number of tie points is large, the dense tie points will affect the detection of the main structure of the building (Figure 9a); if the number of tie points is sparse, it is also difficult for scattered tie points to gather together to reflect the building (Figure 9b). The method proposed in this paper alleviates these issues by reducing the number of redundant features while increasing the number of structural feature points so that the building outline can be better maintained and will be clearly visible (Figure 9c), which is more conducive to the subsequent dense point cloud reconstruction step.

### 4.3. Quantitative Evaluation and Analysis

The feature matching time, feature matching rate and average reprojection error are further used to quantitatively verify the rationality and effectiveness of the proposed method.

The feature matching rate refers to the ratio of the number of successfully matched feature points (more than 2 repeated matches) to the total number of feature points extracted from the image. The mathematical function is shown in Equation (7).

(7) $$\varepsilon \;=\;\frac{N_{\mathrm{match}}}{N_{\mathrm{all}}}$$

where *N_all_* is the total number of feature points extracted from an image and *N_match_* is the number of feature points with more than 2 repeated matches.

The matching time and feature matching rates of the SIFT method, SURF method [39], Wu method and the method proposed in this paper for a 40-km^2^ area are shown in Table 3.

Table 3 shows that there are a large number of feature points in the experimental area, and the original SIFT method takes nearly 20 days to perform feature matching. The SURF method performs faster than SIFT method, and it takes 6 days 23 h to complete the feature matching, which accounts for 34.97% of the time in the SIFT method. After feature point selection by the Wu method, the matching time is significantly reduced to only 14 h 34 min, which accounts for 3% of the initial feature matching time. The method proposed in this paper had a slightly higher matching time than the Wu method, but it still took only 3.5% of the initial feature matching time. In addition, the initial feature points are directly used for feature matching, and the feature matching rate is 68.41%; after using the Wu method for feature point selection, the feature matching rate increased to 80.19%, which verifies that the feature points in the higher Gaussian level are more stable than those in the lower Gaussian levels. The matching rate was increased to 83.42% by using the method proposed in this paper to select the feature points, indicating that the proposed method further improves the stability of the feature points.

The average reprojection error refers to the square root of the ratio of the sum of squared reprojection errors of the sparse point cloud to the total number of tie points after the feature points are processed by aerial triangulation. The mathematical function is shown in Equation (8).

(8) $${\mathrm{RMSE}}_{\mathrm{error}}=\sqrt{\frac{\sum_{i=0}^{N}\left(r_{\mathrm{xi}}^{2}{+r}_{\mathrm{yi}}^{2}\right)}{N}}$$

where *N* is the total number of tie points in the sparse point cloud and $r_{xi}$ and $r_{yi}$ are the reprojection errors of the sparse point cloud in the *x* and *y* directions.

The average reprojection errors of the Wu method and the method proposed in this paper in a 0.75 km^2^ area and 40 km^2^ area are shown in Table 4.

Table 4 reveals that in a small area (0.75 km^2^), the average reprojection error corresponding to the method proposed in this paper is 0.26 pixels, which is lower than the error of the Wu method, indicating that the feature points extracted by the proposed method are more stable and robust. In the larger area (40 km^2^), the average reprojection errors of the Wu method and the method proposed in this paper are 0.28 and 0.34 pixels, respectively. Both methods had slightly higher average reprojection errors in the larger area relative to the smaller area, but the increase was only 3.8%. Furthermore, the error value of the method proposed in this paper was still lower than that of the Wu method, indicating that the former has a higher accuracy for aerial triangulation.

To test the sensitivity of the parameters in this paper for the feature points selection, three image pairs with vegetation area <30%, 30%~70%, and >70% were selected for experiments. These three image pairs represent three different regions with a large number of, moderate and a small number of stable, feature points. The average reprojection errors of these three regions are shown in Table 5.

Table 5 demonstrates that the average reprojection error gradually decreases from image pair A to image pair C, indicating that the proposed method performs better in the region with more stable feature points. In addition, in image pair A, the difference between the average reprojection errors of the proposed method in this paper and the Wu method is 0.02, and this value changes to 0.07 in image pair C, showing that in the region with few stable feature points, the filtering effects of the two methods are similar, while in the region with more feature points, the proposed method is more effective and reliable.

## 5. Discussion and Conclusions

Exploring a robust and efficient feature point selection algorithm to speed up feature matching and ensure matching quality is very important for SfM-based 3D reconstruction. Based on the existing studies, an improved method for stable feature point selection in SfM considering image semantic and structural characteristics is proposed in this paper. Experiments were conducted using real oblique aerial photography data to validate the rationality and effectiveness of the proposed method, and the following conclusions are drawn.

(1) In terms of sparse point cloud reconstruction, compared with the advanced Wu method, the method proposed in this paper increases the feature points in areas with obvious structure features, such as boundary objects and building structure lines, so that the method proposed in this paper can better characterize the scene structure with a small number of feature points.
(2) In terms of the feature matching time, after feature point selection by the Wu method and the proposed method, the matching time is significantly reduced, taking 3% and 3.5% of the initial feature matching time for these two methods, respectively.
(3) In terms of the feature point matching rate, compared with the advanced Wu method, the feature point matching rate of the method proposed in this paper is increased to 83%, which reveals that the feature points selected by this method have a higher stability and robustness.
(4) In terms of the average reprojection error, for the small experimental area (0.75 km^2^), the average reprojection error corresponding to the method proposed in this paper is 21% lower than that of Wu method, and for the large experimental area (40 km^2^), this error is reduced by 20%, indicating that the proposed method achieved a higher aerial triangulation accuracy.

The limitations of our study and future research are mainly focused on the following three aspects. First, although in most cases the feature points obtained by the proposed method in this paper are evenly distributed in the image, in some rare cases, such as the vegetation area occupying a large proportion of the image, the spatial distribution of feature points will be unreasonable. In subsequent research, the classic Harris/Plessey corner detector or Förstner interest operator should be combined with the SIFT descriptor and the proposed method in this paper to further improve the quality of scene representation. Second, improving the efficiency of feature matching is one of the main goals of this study. At present, the method in this paper mainly focuses on the fundamental and core part of algorithm efficiency by making use of CPU calculation in a single machine. In the future, it can be changed into a parallel computing algorithm via multiple machines or multiple GPUs [40,41], and the matching efficiency will be further improved. Finally, the threshold of the number of feature points selection is set to 8192, which is an empirical value obtained from the existing literature. In fact, the setting of this value has a direct relationship with the image size and resolution. In the follow-up research, systematic experiments are needed to explore the rules of threshold setting.

## Figures and Tables

**Table 1:** Classification of feature points.

Type	Type I	Type II	Type III
Classification value	$1\;<\;f\left(i\right)\;\leq \;$2	$0\;<\;f\left(i\right)\;\leq \;$1	0
Meaning	Line-Feature and non-vegetation area	Line-Feature and vegetation area Non-line-Feature and non-vegetation area	Non-line-Feature and vegetation area

**Table 2:** Details of the oblique image data.

Image Size/Pixels		Pixel Size/μm	Focal Length/mm		Number of Images		Altitude/m	Image Overlap/%	
VV*	SV*	VV and SV	VV	SV	VV	SV	680	VV	SV
11,674 × 7514	8900 × 6650	6	120	82	2359	9436		80	80

*: Vertical-view is abbreviated as VV; side-view is abbreviated as SV.

**Table 3:** Statistical results of the feature matching times and matching rates.

	Method	SIFT Method	SURF Method	Wu Method	Proposed Method
Index					
Matching time		19 d* 21 h* 44 min*	6 d 23 h 5 min	14 h 34 min	16 h 38 min
Matching rate		68.41%	71.12%	80.19%	83.42%

* d = days, h = hours and min = minutes.

**Table 4:** Statistical results of the average reprojection errors.

Experimental Area	Method	Average Reprojection Error (Pixels)
0.75 km^2^ (248 images)	Wu method	0.33
	Proposed method	0.26
40 km^2^ (11,795 images)	Wu method	0.34
	Proposed method	0.28

**Table 5:** Statistical results of the average reprojection errors in three image pairs.

Experimental Area	Method	Average Reprojection Error (Pixels)
Image pair A (Vegetation area accounts for 82%)	Wu method	0.38
	Proposed method	0.36
Image pair B (Vegetation area accounts for 51%)	Wu method	0.34
	Proposed method	0.29
Image pair C (Vegetation area accounts for 25%)	Wu method	0.33
	Proposed method	0.26

## Data Availability

Data available on request due to privacy restrictions.
